# The effects of chloroquine and hydroxychloroquine on ACE2 related coronavirus pathology and the cardiovascular system: An evidence based review

**DOI:** 10.1093/function/zqaa012

**Published:** 2020-07-27

**Authors:** Li Chen, Haiyan Chen, Shan Dong, Wei Huang, Li Chen, Yuan Wei, Liping Shi, Jinying Li, Fengfeng Zhu, Zhu Zhu, Yiyang Wang, Xiuxiu Lv, Xiaohui Yu, Hongmei Li, Wei Wei, Keke Zhang, Lihong Zhu, Chen Qu, Jian Hong, Chaofeng Hu, Jun Dong, Renbin Qi, Daxiang Lu, Huadong Wang, Shuang Peng, Guang Hao

**Affiliations:** z1 Georgia Prevention Institute, Department of Medicine, Medical College of Georgia, Augusta University, Augusta, GA, USA; z2 Guangzhou Center for Disease Control and Prevention, Guangzhou, China; z3 Guangzhou First People's Hospital, The Second Affiliated Hospital of South China University of Technology, Guangzhou, China; z4 Department of Gastroenterology, The First Affiliated Hospital of Jinan University, Guangzhou, China; z5 Department of Pulmonary and Critical Care Medicine, The First Affiliated Hospital of Jinan University, Guangzhou, China; z6 Center for Scientific Research and Institute of Exercise and Health, Guangzhou Sports University, Guangzhou, China; z7 Department of urology, The First Affiliated Hospital of Jinan University, Guangzhou, China; z8 Department of Hepatobiliary and Pancreas Surgery, The First Affiliated Hospital Of University of South China, Hengyang, China; z9 Department of Pathophysiology, School of Medicine, Jinan University, Guangzhou, China; z10 Department of Pathophysiology, Key Laboratory of State Administration of Traditional Chinese Medicine of the People’s Republic of China, School of Medicine, Jinan University, Guangzhou, China; z11 Department of Epidemiology, School of Medicine, Jinan University, Guangzhou, China

**Keywords:** coronavirus, angiotensin-converting enzyme 2, chloroquine, hydroxychloroquine, cardiovascular system

## Abstract

The ongoing pandemic of coronavirus disease 2019 (COVID-19) caused by the severe acute respiratory syndrome coronavirus 2 (SARS-CoV-2) poses a serious threat to global public health and there is currently no effective antiviral therapy. It has been suggested that Chloroquine (CQ) and hydroxychloroquine (HCQ), which were primarily employed as prophylaxis and treatment for malaria, could be used to treat COVID-19. CQ and HCQ may be potential inhibitors of SARS-CoV-2 entry into host cells, which is mediated via the angiotensin-converting enzyme 2 (ACE2), and may also inhibit subsequent intracellular processes which lead to COVID-19, including damage to the cardiovascular system. However, paradoxically, CQ and HCQ have also been reported to cause damage to the cardiovascular system. In this review, we provide a critical examination of the published evidence. CQ and HCQ could potentially be useful drugs in the treatment of COVID-19 and other ACE2 involved virus infections, but the antiviral effects of CQ and HCQ need to be tested in more well-designed clinical randomized studies and their actions on the cardiovascular system need to be further elucidated. However, even if it were to turn out that CQ and HCQ are not useful drugs in practice, further studies of their mechanism of action could be helpful in improving our understanding of COVID-19 pathology.

## Introduction

The coronavirus disease 2019 (COVID-19) is due to infection by the severe acute respiratory syndrome coronavirus 2 (SARS-CoV-2).[1-3] The most common symptoms of COVID-19 are fever and cough.[4, 5] Both human-to-human and asymptomatic transmission have been reported.[6] The COVID-19 pandemic has rapidly evolved into a global health crisis as there is currently no proven drug for treating coronavirus patients. However, the strategy of drug repurposing may offer hope for a new approach to COVID-19 treatment.

Among the myriad existing drugs that are potential repurposing candidates for treating COVID-19, the immunomodulatory agents Chloroquine (CQ) and hydroxychloroquine (HCQ) have captured great attention. CQ and its more soluble and less toxic metabolite HCQ are primarily used for prophylaxis and treatment of malaria, but they have also been reported to effectively inhibit the effects of certain viruses, such as severe acute respiratory syndrome coronavirus (SARS-CoV) and influenza A H5N.[7-10] Recently, the possible use of CQ/HCQ as a repurposed therapeutic agent against COVID-19 has been explored.[11]

Angiotensin-converting enzyme 2 (ACE2), a new homolog of ACE, can convert angiotensin II (Ang II) to Ang(1-7)[12, 13]. Ang(1-7) binds and activates the G-protein coupled receptor Mas (MasR)[14] and acts as a natural damping mechanism for the activation of the classical renin-angiotensin system (RAS),[12, 13, 15, 16] which plays a critical role in maintaining normal cardiovascular (CV) functions. Apart from its crucial role in CV disease, ACE2 has also been shown to be a functional host cellular entry receptor for coronavirus that directly binds the viral spike (S) protein, which is primed by the transmembrane serine protease 2 (TMPRSS2).[17-20]

The ongoing COVID-19 pandemic, caused by SARS-CoV-2, poses a serious threat to global public health, and cross-sectional data suggest that SARS-CoV-2 infected patients have a high prevalence of CV disease.[21, 22] Recent data indicated that CQ and

HCQ (CQ/HCQ) may have a promising ability to inhibit SARS-CoV-2 and other ACE2 related viral diseases [7-9], but the effects of CQ/HCQ on the CV system seem paradoxical. CQ/HCQ shows CV benefits, including a reduction in the risk of developing hyperlipidemia and diabetes mellitus, but CV disorder has also been reported as one of the rare but severe side effects of CQ/HCQ.[23]

In this review, we summarize and evaluate the published evidence concerning the actions and mechanisms of action of CQ/HCQ in treating SARS-CoV-2 and other ACE2 related viral infections. We conclude that further mechanistic studies as well as well-designed clinical randomized trials are needed to investigate the molecular pathogenesis of SARS-CoV-2 infection and to examine the antiviral efficacy of CQ/HCQ against COVID-19. Furthermore, the effects and mechanisms of action of CQ/HCQ on the CV system should be further investigated.

**ACE2 and its role in viral infection**

The RAS is a humoral regulation cascade that elegantly orchestrates key vascular physiology in humans. SARS-CoV-2 infection has been proposed to interfere with RAS through the ACE2 receptor for host cell entry (**Figure 1**).[19, 24] Severe COVID-19 infection has many clinical characteristics which are strikingly similar to the effects of overactivation of the RAS. It has been reported that coagulation is activated and accelerated in patients with SARS-CoV-2.[25] The complex entry process of coronavirus into susceptible cells requires multistep actions of receptor-binding and proteolytic processing of the S protein to promote virus-cell fusion. S protein cleavage occurs at the boundary between the S1 and S2 subunits, and S is further cleaved at the S2' site by host proteases to facilitate the fusion of viral and cellular membranes via extensive irreversible conformational changes.[26-29] A recent study provided fresh evidence that SARS-CoV-2 exploits ACE2 and TMPRSS2 for host cell entry.[24] Like SARS-CoV entry into host cells,[17] the S glycoprotein domain B ($S^B$) of SARS-CoV-2 binds to the human ACE2 (hACE2) receptor and is subsequently primed by TMPRSS2.[30, 31] Moreover, SARS-CoV-2 S has a similar or even higher (~10- to 20-fold) affinity for binding to hACE2 as compared to SARS-CoV S.[30, 31] However, a novel and very important feature of SARS-CoV-2 S is that it harbors a furin cleavage site at the S1/S2 boundary, which is processed during biosynthesis.[31] Therefore, the presence of the polybasic cleavage site in SARS-CoV-2 S, processed by furin-like proteases, may modulate tropism, transmissibility and pathogenicity of SARS-CoV-2, making it a highly pathogenic virus, like avian influenza viruses.[32] The relationship between the expression level of ACE2 and susceptibly to SARS-CoV-2 infection still remains elusive. It will thus be interesting to determine whether SARS-CoV-2 interferes with ACE2 expression and activity as well as to evaluate the functional consequence of the potential cleavage site used in SARS-CoV-2 and its impact on transmissibility and pathogenesis in animal models.

In order to better understand the initial step of SARS-CoV-2 infection, elucidation of the interactional mechanism between the receptor-binding domain (RBD) of SARS-CoV-2 S and ACE2 appears to be particularly important. Two recent independent studies have reported the cryo-EM structure of the SARS-CoV-2 spike trimer.[30, 31] Moreover, another study presented the cryo-EM structures of the full-length hACE2-$B^0AT1$ (the neutral amino acid transporter) complex and a complex between the RBD of SARS-CoV-2 and the hACE2-$B^0AT1$ complex as well as the hACE2-RBD interface.[33] In addition, analytical modelling of structure predicted the potential residues of SARS-CoV-2 RBD that are recognized by ACE2.[34] Furthermore, X-ray crystallography data at a higher resolution showed the interaction between SARS-CoV-2 RBD and ACE2, demonstrating that SARS-CoV-2 and SARS-CoV RBD share high structural similarity.[35] It remains to be investigated how SARS-CoV-2 alters the conformations of S glycoprotein trimers and the interactions between ACE2 and S proteins in receptor-mediated endocytosis. Interestingly, single-cell RNA-sequencing data from multiple healthy human tissues discovered that the SARS-CoV-2 entry receptor ACE2 and the viral entry-associated protease TMPRSS2 are highly expressed in nasal goblet and ciliated cells.[36] These new insights indicate that the primary viral SARS-CoV-2 transmission occurs through infectious droplets. Although TMPRSS2

activity is essential for viral transmission, it still needs to be determined whether the endosomal cysteine proteases cathepsin B and L or other proteases, as reported in SARS-CoV and Middle East respiratory syndrome coronavirus (MERS-CoV),[26, 28, 37-39] are involved in priming SARS-CoV-2 S. Hence, further mechanistic studies are needed to elucidate the underlying detailed mechanism of SARS-CoV-2 entry into host cell and to test the potential of SARS-CoV-2 neutralizing antibodies.[40, 41]

Several studies have reported that 3% to 29% of COVID-19 patients develop acute respiratory distress syndrome (ARDS) which is a common complication and cause of death as a result of SARS-CoV-2 infection.[4, 5, 21, 22] Although the pathophysiology of COVID-19 has not been completely unraveled, the potential main mechanism of COVID-19-asscoiated ARDS would appear to be the immune-pathological event of the so-called cytokine storm. Laboratory tests showed that patients infected with SARS-CoV-2 express high amounts of pro-inflammatory cytokines and chemokines, including interleukin (IL)-1□, tumor necrosis factor α (TNFα), interferon-γ (IFN-γ), C-X-C motif chemokine ligand (CXCL)-10, and monocyte chemoattractant protein 1 (MCP1).[4] The evidence obtained from the postmortem biopsy study of a 50-year-old male patient suggested that the severe immune injury in COVID-19-associated ARDS is related to over-activation of T cells, manifested by the elevation of T-helper-17 (Th17) and high cytotoxicity of CD8 T cells.[42] SARS-CoV-2, SARS-CoV and MERS-CoV cause acute lethal disease characterized by dysregulated and excessive immune responses and lung damage during viral infection. It was reported that relative delayed type I interferon (IFN-I) signaling promoted inflammatory monocyte-macrophage accumulation in BALB/c mice infected with SARS-CoV.[43] Consequently, these accumulated mononuclear macrophages produce more monocyte chemoattractants through activating the IFN-α/β receptors and mononuclear macrophage-derived pro-inflammatory cytokines, such as TNFα, IL-1β and IL-6, induce apoptosis of T cells. Robust virus replication and excessive inflammatory responses induced the release of IFN-α/β and IFNγ, causing inflammatory cell infiltration via Fas–Fas ligand (FasL) signaling or tumor necrosis factor-related apoptosis-inducing ligand (TRAIL)–death receptor 5 (DR5) signaling. This eventually results in the apoptosis of airway and alveolar epithelial cells.[44-46] The apoptosis of these endothelial and epithelial cells could potentially lead to vascular leakage and alveolar edema, which is regarded as playing a key role in the pathogenesis of virus infection-associated ARDS. It seems to be this deadly uncontrolled cytokine storm that triggers the frantic attack on the body by the immune system causing ARDS and finally unprecedented mortality in severe cases of SARS-CoV-2 infection. Future work needs to investigate the details of the IFN signaling involved in SARS-CoV-2 infection, how the inflammatory response is triggered as well as the type of cell death that occurs during COVID-19. Also, further autopsy or biopsy studies, including more patients of different ages and backgrounds, would be needed to examine the histopathological changes and ACE2 levels in different tissues.

**CQ/HCQ actions on pH**

The acid milieu in endosomes and lysosomes (pH between 5 and 6) is due to a bafilomycin-sensitive pump that concentrates $H^+$ in the lumen of endosomes/lysosomes.[47] This low pH is essential for virus/cell membrane fusion.[8, 48-51]. CQ was reported to cause an increase in the intra-lysosomal pH of macrophages.[52] However, there is still no evidence showing the effect of HCQ on the pH dynamics of endosomes/lysosomes. Nevertheless, both CQ and HCQ are weak bases so they should both be able to elevate endosomal pH and could thereby inhibit virus/cell membrane fusion. It has recently been reported that CQ is highly effective in the control of SARS-CoV-2 infection *in vitro*.[11] Compared to remdesivir (GS-5734), the time-of-addition assay showed that CQ functioned at entry as well as at post entry stages of the SARS-CoV-2 infection in Vero E6 cells.[11] Similarly, another *in vitro* study also found that HCQ can efficiently inhibit SARS-CoV-2 infection via the same routes.[53] The therapeutic effect of both CQ and HCQ may be due to blockade of the transport of SARS-CoV-2 from early endosomes (EEs) to endo-lysosomes (ELs), which seems to be the same viral genome releasing mechanism that operates in the case of SARS-CoV. However, the mode of actions of CQ and HCQ showed discrepancy in certain aspects such as in the morphology and pH values of endosomes/lysosomes.[53] Another recent study found that HCQ exhibited a smaller $EC_{50}$ than CQ in *in vitro* anti-SARS-CoV-2 activity and physiologically-based pharmacokinetic models indicated that HCQ is likely to be more effective than CQ in the treatment of SARS-CoV-2 infection.[54] However, there are controversies about the effect of CQ/HCQ in altering endosomal /lysosomal pH and treating viral infection. It was reported that CQ/HCQ could directly bind to nucleic acids and inhibit the activation of the endosomal toll-like receptor (TLR) by masking TLR ligand-binding epitopes rather than increase the endosomal pH.[55] In addition, CQ was shown to inhibit autophagy mainly through impairing autophagosome fusion with lysosomes rather than by increasing pH in this organelle.[48] Furthermore, there was a study showing that CQ could enhance porcine circovirus 2 infection of porcine epithelial cells via inhibition of endosome-lysosome system acidification.[56] The effect of CQ/HCQ may differ from cell type to cell type and between virus species. Nevertheless, whether CQ/HCQ are able to affect the acidity of EEs and ELs in SARS-CoV-2 infection should be examined carefully in the future.

The progressive acidification that normally occurs from EEs to ELs depends on a high $Ca^{2+}$ concentration in the EEs. The pH in the lumen of these organelles decreases in line with a decrease in the $Ca^{2+}$ concentration.[56] $Ca^{2+}$ signaling has been demonstrated to be involved in viral fusion into host cells of many viruses such as Ebola virus (EBOV), MERS-CoV and SARS-CoV.[57] $Ca^{2+}$ release from intracellular stores within the endolysosomal system, via two-pore channels (TPC1, TPC2) and channels belonging to the mucolipin family (e.g. TRPML1) can be evoked by nicotinic acid adenine dinucleotide phosphate (NAADP) and phosphatidylinositol 3,5-bisphosphate ($PIP_2$).[57, 58] In pancreatic acinar cells, antibodies against TPC2 are very effective, and much more effective than antibodies against TPC1, in reducing NAADP-elicited $Ca^{2+}$ release from acidic stores.[59] In this context it is of particular interest that it has very recently been shown that blocking TPC2 activity by tetrandrine decreases entry of SARS-COV-2 S pseudovirions.[60] In contrast, a TRPML1 inhibitor had no effect.[60] The endo-lysosomal $Ca^{2+}$ level and pH, altered by TPC activity,[61-63] regulate the activity of furin required for proteolytic activation of the S protein and viral fusion.[64, 65] It was reported that inhibition of TPCs rather than TRPML1 could block MERS-CoV infectivity.[66] Moreover, it has been demonstrated that the endosomal calcium channels TPC1 and TPC2 are necessary for EBOV infection.[57] Interestingly, tetrandrine has been identified as a highly potent and low cytotoxic TPCs inhibitor.[57] Because of its ability to disrupt TPCs function, tetrandrine can prevent EBOV from escaping the endosomal network into the cell cytoplasm and thus block EBOV infection. Some other $Ca^{2+}$ channel blockers such as amiodarone, verapamil, nimodipine, diltiazem, bepridil and lomerizine could effectively protect against filoviral entry into target cells.[67, 68] Since these calcium channels are responsible for controlling trafficking and translocation of endosomes containing virus particles, it would be potentially intriguing to explore the effect of TPCs in SARS-CoV-2 infectivity and to screen $Ca^{2+}$ channel blockers for their effects in halting SARS-CoV-2 infection.

Although an open-label non-randomized clinical trial with a small sample size showed that HCQ treatment is significantly associated with SARS-CoV-2 load reduction/disappearance in COVID-19 patients,[69] there are several serious limitations in that study. Furthermore, a multicenter prospective observational study reported that CQ has the potential to shorten the time to SARS-CoV-2 viral suppression and duration of fever in patients with moderate symptoms at earlier stages of the disease.[70] Another open label, randomized controlled trial did not show additional benefits of virus elimination from adding HCQ to the current standard of care in patients with mainly persistent mild to moderate COVID-19.[71] Meanwhile, a retrospective analysis of 368 cases with confirmed SARS-CoV-2 infection indicated that using HCQ, either with or without azithromycin, could not reduce the risk of needing mechanical ventilation in patients hospitalized with COVID-19.[72] Fortunately, at least 14 clinical trials are already registered in the clinicaltrials.gov[73] to evaluate the effects of CQ/HCQ to SARS-CoV-2.

**CQ/HCQ relationships to ACE2**

There is much evidence indicating that SARS-CoV and SARS-CoV-2 infect host cells through ACE2.[17, 18, 74, 75,60] Furthermore, CQ could block SARS-CoV fusion with and entry into the host cell through interfering with the glycosylation of the ACE2 receptor and the S protein.[8] CQ/HCQ have promising ability to inhibit SARS-CoV-2 and ACE2-related viral infection. It has been shown that CQ is an effective inhibitor of the replication of the SARS-CoV in Vero E6 cell culture.[76] Another study further confirmed that CQ is effective against SARS-CoV in Frankfurt and Urbani strains.[8] In addition, this study found that CQ impaired the terminal glycosylation of ACE2, suggesting that the variations in its glycosylation status might result in the ACE2-SARS-CoV interaction being less efficient and therefore inhibit virus entry when the cells are treated with CQ. Also, it was shown that the recombinant SARS-CoV S protein downregulates ACE2 expression.[77]

In experimental mouse models, infection with avian influenza A H5N1 virus resulted in down-regulation of ACE2 expression in the lung.[78] Genetic inactivation of ACE2 caused severe lung injury in H5N1-challenged mice, suggesting a role for ACE2 in H5N1-induced lung pathologies.[78] CQ was found to effectively inhibit autophagy in the lungs of avian influenza H5N1 mice and to ameliorate the acute lung injury and further, significantly improve the survival rate in mice infected with live avian influenza A H5N1 virus.[9] There is also evidence that CQ had an inhibitory effect against the replication of human influenza A virus H1N1 and H3N2 *in vitro*.[7] ACE2 could mediate the severe acute lung injury induced by influenza A (H7N9) virus infection in an experimental mouse model. Moreover, ACE2 deficiency worsened the disease pathogenesis markedly, mainly by targeting the Angiotensin II receptor type 1 (AT1 receptor, AT1R).[79] Therefore, the potential effects and mechanism of CQ/HCQ against ACE2 related viruses appears worth further investigation.

**Paradoxical effect of CQ/HCQ in the cardiovascular system**

CQ/HCQ shows CV benefits, including reductions in the risks of developing hyperlipidemia, diabetes mellitus, and thrombosis, as well as improving insulin sensitivity, glucose profiles, and HbA1c, and decreasing cholesterol, triglycerides, and low-density lipoprotein-cholesterol (LDL-c).[80, 81] CQ/HCQ is extensively used in the treatment of rheumatic diseases, the patients of which are at higher risk of CV disease.[82, 83] A retrospective study of a cohort of 1,266 patients with rheumatoid arthritis (RA) found that HCQ was associated with an approximately 72% reduction in the risk of CV disease.[84] Another longitudinal registry showed that, compared to non-users, RA patients with HCQ treatments had significantly lower levels of total and low-density cholesterol.[85] A longitudinal cohort study of 264 systemic lupus erythematosus patients found lowered serum cholesterol levels associated with HCQ treatment.[86] A prospective, multicenter observational study of 4905 adults with RA also reported that use of HCQ is associated with a 38% reduction of diabetes risk.[87] HCQ is also found to reduce blood pressure variability among 899 systemic lupus erythematosus patients.[88]

There are a few small clinical trials studying the effects of CQ/HCQ on CV risks in humans. A randomized controlled trial (RCT) carried out among 116 patients with metabolic syndrome found that a one-year CQ treatment decreased blood pressure, lipids, and the activation of c-Jun N-terminal kinase (JNK).[89] Another randomized, double-blinded, placebo-controlled crossover study found that a 8-week HCQ treatment decreased insulin resistance, total cholesterol, and LDL-c among 23 RA patients.[90] A small open-label clinical trial administered HCQ to 13 obese participants for six weeks, which significantly increased the insulin sensitivity index.[91] Another RCT with 135 patients with sulfonylurea-refractory type 2 diabetes proved that HCQ could decrease glycated hemoglobin and improve glucose tolerance.[92]

In animal studies, CQ could lower blood pressure through Toll-like receptor signaling and prevent the subsequent recruitment of immune cells to the vasculature in spontaneously hypertensive rats.[93] In rat hepatocytes, CQ was shown to be an effective inhibitor of cholesterol synthesis.[94] It has also been reported that CQ improved the cardiac diastolic function by inhibiting autophagy in streptozotocin-induced heart failure with preserved ejection fraction in mice.[95] Evidence was also found that activation of ataxia telangiectasia mutated with low-dose CQ decreased features of the metabolic syndrome including atherosclerosis in mice.[96] Taken together, the claimed CV benefits of CQ/HCQ are mostly generated from animal studies or observational studies in humans.

In rare cases, CQ/HCQ treatment presents cardiotoxicity including hypotension, arrhythmia, atrioventricular block[97], cardiomyopathy[23, 98] and heart failure[23, 99], which could be serious.[100, 101] The cardiotoxicity of CQ/HCQ may be under-recognized[23]. Among the 25 episodes of intentional CQ overdosage, 19% died, and 50% had cardiac arrest.[102] *Ex vivo* acute CQ treatment decreased heart function, and *in vivo* chronic low-dose CQ treatment significantly decreased aortic output and total work in hearts.[103] A systemic review of  patients with cardiac complications attributed to CQ/HCQ found that for the 78 patients reported to have been withdrawn from CQ/HCQ treatment, 44.9% recovered normal heart function, while 12.9% had suffered irreversible damage and 30.8% died.[101]

The mechanisms underlying the effects of CQ/HCQ on the CV system are not fully understood (**Figure 2**). CQ could improve insulin sensitivity by increasing the affinity of insulin receptors, inhibiting insulin degradation, and increasing insulin secretion.[104] CQ/HCQ could also increase the lipid clearance rate and expression of LDL receptors.[105] HCQ is thought to protect against accelerated atherosclerosis, targeting toll-like receptor signaling, cytokine production, T-cell and monocyte activation, oxidative stress, and endothelial dysfunction.[106] HCQ can also reduce the induction of endosomal NADPH oxidase (NOX) by TNFα, IL-1β and antiphospholipid antibodies (aPL) through the inhibition of the translocation of the catalytic subunit of NOX2 into the endosome, which is involved in many inflammatory and pro-thrombotic signaling pathways.[107] However, chronic use of CQ/HCQ can result in an acquired lysosomal storage disorder, leading to cardiomyopathy characterized by concentric hypertrophy and conduction abnormalities associated with increased adverse clinical outcomes and mortality.[108] HCQ is structurally and mechanistically similar to the class IA

antiarrhythmic quinidine,[109] and may therefore inhibit voltage-gated sodium and potassium channels, prolonging the QT interval and increasing the risk of 'torsades de pointes' (a specific type of abnormal heart rhythm) and sudden cardiac death.[110] An animal study found that high-dose CQ significantly impaired mitochondrial antioxidant buffering capacity and accentuated oxidative stress and mitochondrial dysfunction in pressure-overload hypertrophy.[103] In addition, CQ may increase CV risk by impairing the terminal glycosylation of ACE2,[8] which possibly amplifies ACE/AngII/AT1 axis signaling and depresses ACE2/Ang1-7/MasR axis signaling.

**Concluding remarks**

The role of ACE2 in the action of CQ/HCQ needs to be further studied. Based on the existing studies, CQ/HCQ may be potential drugs for treatment of COVID-19 and other ACE2 - related virus infections. However, the use of CQ/HCQ should be dealt with cautiously and careful monitoring of potential cardiotoxicity is required in clinical practice and research. The use of high doses and long-term CQ/HCQ treatment require particular care and serious consideration.

Understanding how the virus enters host cells, and the details surrounding how it binds to the receptor on the host cell, are critical for facilitating the development of detection methods, antiviral therapeutics, and vaccines. Reliable information on the molecular mechanisms underlying viral entry and proliferation will enable us to target and combat the virus.

Finally, it is challenging to interpret the extensive amount of COVID-19 related research that has been published within a very short space of time. This is a highly unusual situation in the routine life cycles of any research topic. We therefore need to maintain a degree of healthy skepticism when interpreting the COVID-19 related scientific literature.

**Search strategy**

We carried out electronic searches using PubMed, Web of Science, Researchgate and Google. The search terms were "virus", "coronavirus", "angiotensin-converting enzyme 2", "chloroquine", "hydroxychloroquine", "cardiovascular system", and others, alone and in combination. Many firstly identified references were investigated further to find the original primary research articles that were then cited in the review.

**Conflict of interest**: none declared.

**Acknowledgments**

H.W. is supported by the National Natural Science Foundation of China (81871542 and 81670359). S.P. is supported by the Medical Scientific Research Foundation of Guangdong Province, China (A2019205 and A2020121) and the Fundamental Research Funds for the Central Universities (21620424).

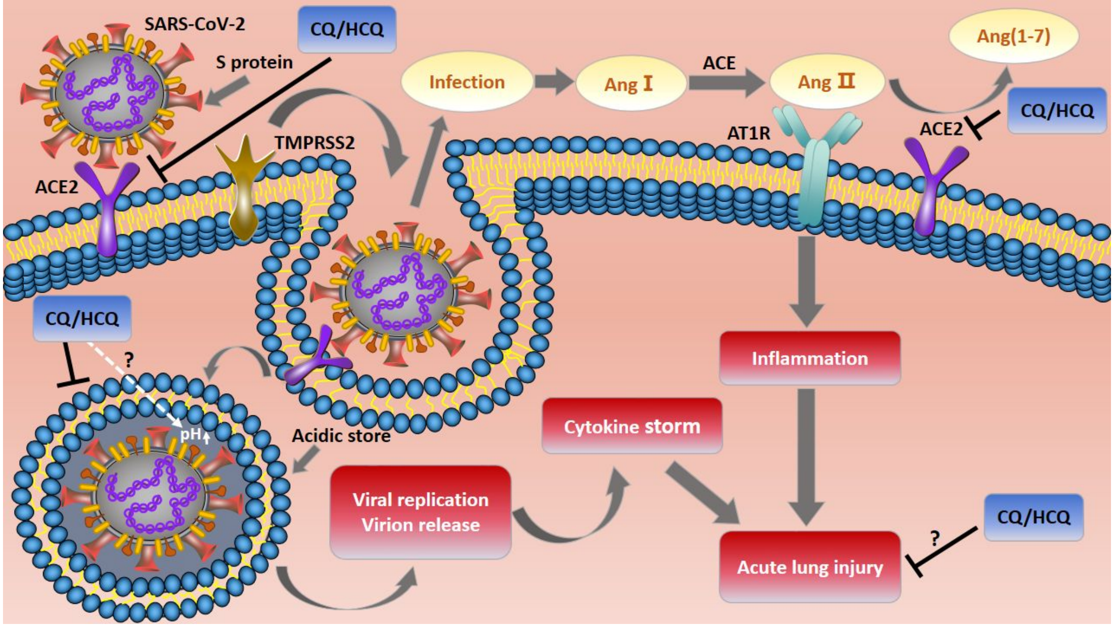

**Figure 1 The effects of chloroquine and hydroxychloroquine on ACE2 related viral infection.** The initial entry of SARS-CoV-2 (an enveloped virus)[111] into host cells depends on ACE2 and TMPRSS2. The S protein of SARS-CoV-2 binds to the functional receptor ACE2 and employs TMPRSS2 for its priming. S protein is cleaved by TMPRSS2 at S2' site which results in virus/membrane fusion.[27] Both ACE2 and TMPRSS2 facilitate the virus transport into the target cell through the early and late endosomes where eventually the viral genome will be released into the cell cytoplasm. SARS-CoV-2 infection could influence the balance of RAS, which leads to Ang II accumulation through the ACE/AngII/AT1R axis and eventually causes acute lung injury. CQ/HCQ may block SARS-CoV-2 fusion with the host cell and entry into the target cell through elevating the pH in the endolysosomal system and/or by interfering with the glycosylation of the ACE2 receptor and the S protein.[8]

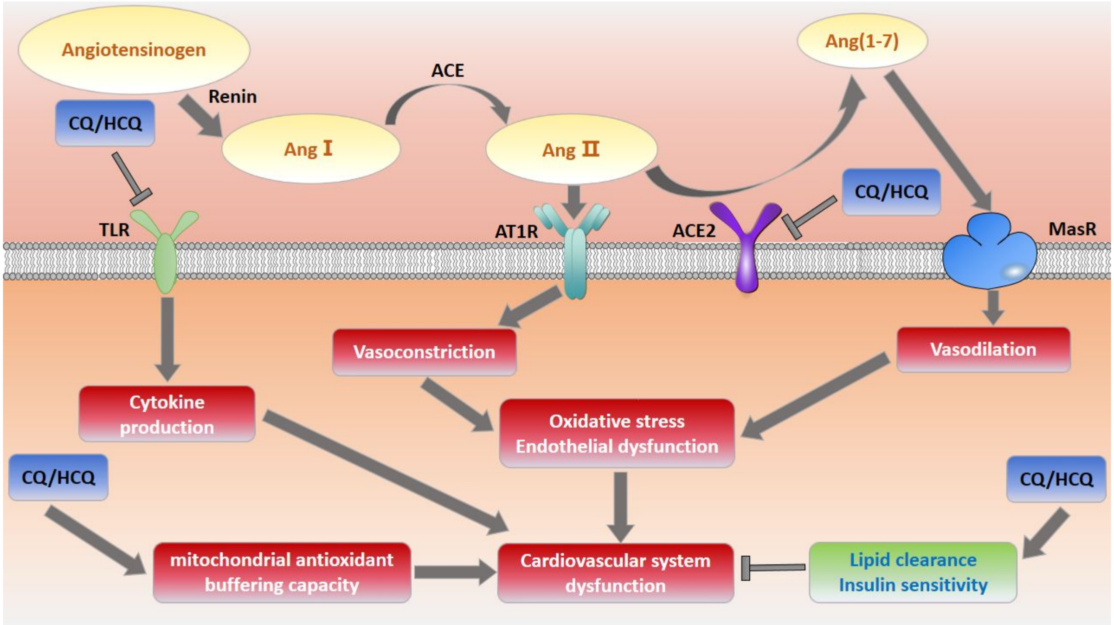

**Figure 2 The effects of chloroquine and hydroxychloroquine on the cardiovascular system.** CQ/HCQ could protect against accelerated atherosclerosis targeting TLR signaling, cytokine production, T-cell and monocyte activation, oxidative stress, and endothelial dysfunction. However, CQ/HCQ interferes with the glycosylation of ACE2 and this leads to dysregulation of the RAS, which eventually causes imbalance of the ACE/AngII/AT1 axis and the ACE2/Ang1-7/MasR axis. Meanwhile, CQ/HCQ can cause cardiotoxicity which may increase the risk of CV disease. Therefore, CQ/HCQ may have a paradoxical effect on the CV system.

ACE2, angiotensin-converting enzyme 2. Ang I, angiotensin I. Ang II, angiotensin II. AT1R, Angiotensin II receptor type 1. CQ, chloroquine.CV, cardiovascular. HCQ, hydroxychloroquine. Mas receptor, MasR. RAS, renin–angiotensin system. SARS-CoV-2, severe acute respiratory syndrome coronavirus 2. S protein: Spike protein. TMPRSS2, transmembrane serine protease 2. TRL, toll-like receptor.
